# Radical Polymerization of Alkyl 2-Cyanoacrylates

**DOI:** 10.3390/molecules23020465

**Published:** 2018-02-20

**Authors:** Cormac Duffy, Per B. Zetterlund, Fawaz Aldabbagh

**Affiliations:** 1 Henkel Ireland Operations & Research Limited, Whitestown, Dublin 24, Ireland; cormac.duffy@henkel.com; 2 School of Chemistry, National University of Ireland Galway, University Road, Galway H91 TK33, Ireland; 3 Centre for Advanced Macromolecular Design (CAMD), School of Chemical Engineering, The University of New South Wales, Sydney, NSW 2052, Australia; p.zetterlund@unsw.edu.au; 4 Present address: Department of Pharmacy, School of Life Sciences, Pharmacy & Chemistry, Kingston University, Penrhyn Road, Kingston upon Thames KT1 2EE, UK

**Keywords:** cyanoacrylate, instant adhesives, super glue, polymerization, radical

## Abstract

Cyanoacrylates (CAs) are well-known fast-setting adhesives, which are sold as liquids in the presence of stabilizers. Rapid anionic polymerization on exposure to surface moisture is responsible for instant adhesion. The more difficult, but synthetically more useful radical polymerization is only possible under acidic conditions. Recommendations on the handling of CAs and the resulting polymers are provided herein. In this review article, after a general description of monomer and polymer properties, radical homo- and copolymerization studies are described, along with an overview of nanoparticle preparations. A summary of our recently reported radical polymerization of CAs, using reversible addition-fragmentation chain transfer (RAFT) polymerization, is provided.

## 1. Introduction 

Alkyl 2-cyanoacrylates or cyanoacrylates (CAs) are a family of vinyl monomers renowned for their high reactivity, instant adhesive properties, and wide-ranging applications [1,2,3,4,5,6]. Short chain CAs, like methyl- and ethyl 2-cyanoacrylate have found great utility as the major components of industrial and household instant adhesives or “super glues”, including those manufactured at Henkel under the Loctite brand (Table 1) [4,5,6].

**Figure 1:**
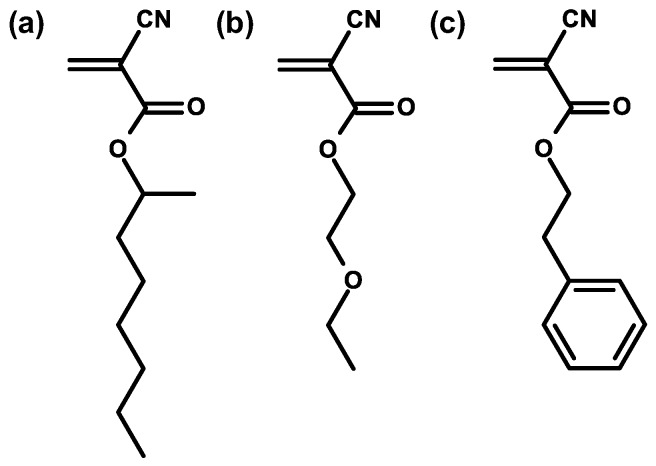
(**a**) 2-octyl (**b**) β-ethoxyethyl (**c**) phenylethyl 2-cyanoacrylates.

Longer-chain CAs, such as *n*-butyl and 2-octyl 2-cyanoacrylate (Figure 1), have found utility as surgical suture replacements for skin and tissue adhesives [7,8,9,10]. Poly(CAs) have gained recognition for numerous biomedical applications due to their favorable biocompatibility, biodegradability, and low toxicity. Colloidal nanoparticles derived from CAs have shown great promise in the field of drug and vaccine delivery [11,12,13,14,15,16]. CA is used in latent fingerprint development in forensic science, whereby CA vapors adheres to trace amino acids present in the fingerprint [17,18]. Due to its unsaturated ester group, allyl 2-cyanoacrylate has the ability to undergo crosslinking reactions at elevated temperatures, a feature that has found use in thermally resistant adhesives [5]. β-Methoxyethyl- and β-ethoxyethyl 2-cyanoacrylate have lower vapor pressures compared to shorter chain CAs and are often used as low odor adhesives. Additionally, these adhesives avoid a phenomenon characteristic of their shorter chain counterparts, known as “blooming”, whereby polymerized monomer vapor is deposited as a fine chalky powder on the surface near an adhesive bond-line, and are sometimes referred to as “low-bloom” adhesives [4]. Phenylethyl 2-cyanoacrylate differs from the previously named CAs in that it is a solid and is utilized in industrial adhesive tapes and films [19].

The general monomer chemistry, synthesis, modes of polymerization, and the physical characteristics of the resulting polymers, along with several toxicological evaluations, have been detailed in a number of reviews [1,2,3,4,5,6,7,8,9,10,11,12,13,14,15,20,21,22]. In this review, after a general overview of reactivity and properties, the focus is the radical polymerization of CAs, with the advantages over the more facile anionic pathway clearly outlined.

## 2. General Reactivity and Properties

### 2.1. Anionic-Type Polymerizations

**Scheme 1:**
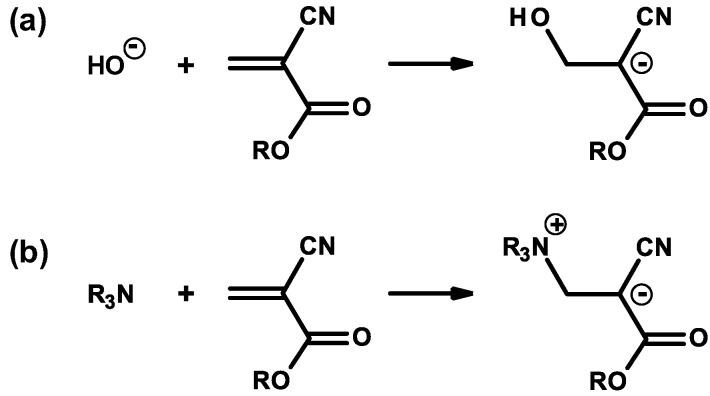
(**a**) Anionic and (**b**) zwitterionic initiation.

The high reactivity of CAs is attributed to the strong electron-withdrawing nitrile (CN) and ester (CO_2_R) groups attached to the α-carbon of the double bond. The β-carbon of CAs is activated towards nucleophiles, such as anions, as well as weak bases, water, and alcohols (Scheme 1). In an adhesive context, polymerization or curing occurs through rapid initiation from the thin film of moisture commonly found on the surface of most materials.

**Scheme 2:**
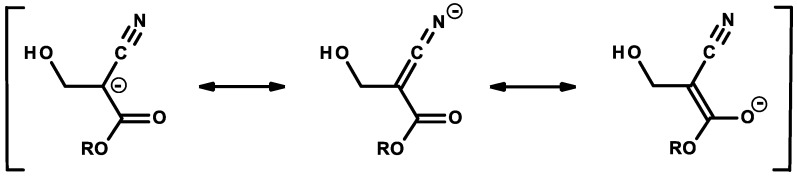
Resonance stabilization of cyanoacrylate (CA) α-carbanion adduct.

Initiation can rapidly occur upon contact with anions, as well as with non-dissociated base species, such as tertiary amines and tertiary phosphines, leading to zwitterionic polymerization [23,24,25,26,27,28]. Regardless of whether polymerization is initiated by an anionic or non-dissociated nucleophilic base, a propagating carbanion is formed on the α-carbon, which is resonance stabilized through the CN and CO_2_R groups (Scheme 2).

**Scheme 3:**
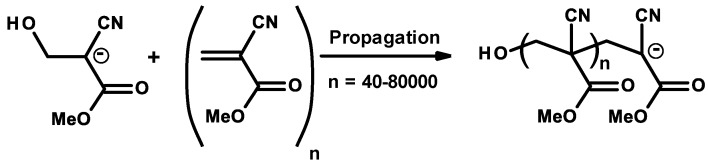
Anionic polymerization: Propagation.

The stabilization of the negative charge through delocalization at the α-carbon atom, in combination with the sterically unhindered and highly electrophilic nature of the β-carbon, makes the cyanoacrylate molecule uniquely reactive. The carbanion adds to another monomer molecule to generate a dimeric species, which in turn reacts with more monomer until high molecular weight polymer is formed, typically in the range of 10^5^–10^7^ g·mol^−1^ (Scheme 3) [29]. The poly(CA) molecular weights are not greatly affected by temperature, but can be affected by pH [30]. Solution polymerizations of *n*-butyl 2-cyanoacrylate in tetrahydrofuran (THF) at 20 °C using tetrabutylammonium salts as initiators gave extremely high rates of polymerization, with propagation rate coefficient (*k*_p_) values close to 10^6^ L·mol^−1^·s^−1^ [28]. The *k*_p_ values for CAs are significantly greater than that for the anionic polymerization of methyl methacrylate (MMA), with *k*_p_ = 775 L·mol^−1^·s^−1^ being initiated by a tetraphenylphosphonium salt under similar experimental conditions of 20 °C in THF [31]. Rapid initiation of CA polymerization leads to characteristics close to those of ideal living polymerization, with molecular weights in approximation to theoretical values, based on the monomer/initiator ratio [32].

**Scheme 4:**
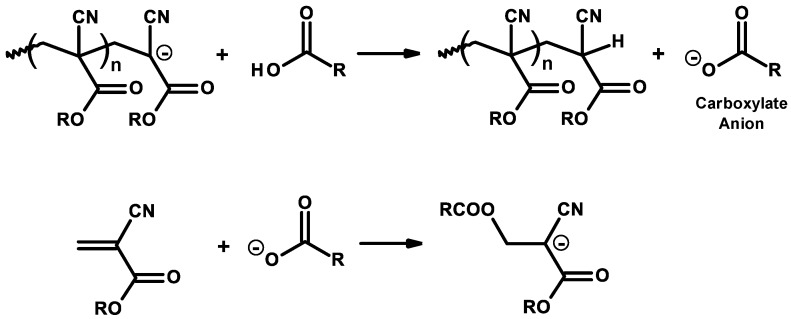
Chain-transfer in anionic polymerization.

**Scheme 5:**
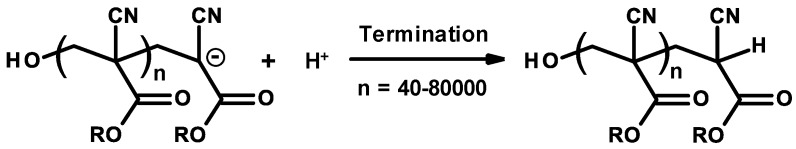
Termination of propagating chain with strong acid.

Propagation of CA polymerization will continue until all available monomers are consumed or until a chain transfer or termination step intervenes. It is believed that some chain-transfer reactions can occur in the presence of weak acids, such as the carboxylic acids formed from the hydrolysis of CA monomers [1]. Transfer of a proton to the propagating carbanion will terminate the growth to produce a dead-chain; however, the resulting conjugate base (carboxylate anion) is able to initiate a new growing polymer chain by addition onto a monomer (Scheme 4). Strong acids will act as chain terminators by protonation of the anion and will rapidly kill the polymerization (Scheme 5). In the case of strong mineral acids (e.g., sulfuric acid), the conjugate base is not nucleophilic enough to initiate further anionic polymerization.

The investigations carried out by Pepper et al. demonstrated that in the absence of strong acid, the polymerization has no intrinsic termination reactions, and the poly(CA) carbanions are stable enough to remain active, even after addition of small amounts of common terminating agents, such as water, oxygen or CO_2_ [23,24,25,26,27,28,32]. The stability of the propagating species is in stark contrast to other common monomers like styrenics or (meth)acrylates, for example, where, in the case of organolithium-initiated anionic polymerizations, the introduction of trace amounts of air or moisture terminates polymer growth by reacting with the active chain-end anions or the (often organometallic) initiator. With the latter conventional anionic polymerizations stringent experimental conditions, including inert atmospheres and glove boxes are a requirement [33,34]. It is noteworthy that the recent advances in the synthesis of well-defined complex macromolecular architectures using anionic polymerizations (including star, comb/graft, cyclic, branched, hyperbranched, dendritic and multi-block multi-component polymers [35]) are yet to be achieved with the highly reactive monomer subject of this review.

### 2.2. Polymer Properties and Stability

The glass transition temperatures (*T*_g_) of common poly(CAs) are shown in Table 2. In general, the *T*_g_ values decrease with increasing ester chain length and steric bulk. *T*_g_ values can, however, vary to some extent depending on the method employed for determination, such as dilatometry and dynamic thermal analysis. Additionally, the means of polymer sample preparation (anionic or radical polymerization), polymerization temperature and resulting molecular weight can impact on *T*_g_ values [36,37].

**Scheme 6:**
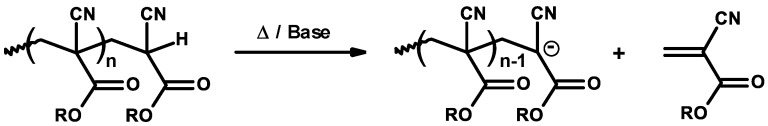
Degradation of poly(CAs).

Poly(CAs) have relatively poor thermal stability [39,40,41,42,43,44,45,46,47,48], and start to degrade slightly above their *T*_g_, which is significantly less than their ceiling temperature (*T*_c_) [42,45]. The latter is the temperature at which the rates of polymerization and depolymerization are equal [49]. Thermal behavior of isolated polymers, however, can be very complex, and degradative reactions other than depolymerization will often occur at temperatures below the *T*_c_. Poly(ethyl 2-cyanoacrylate), for example, begins to degrade at around its *T*_g_ of approximately 150 °C, whereas its *T*_c_ has been measured as 276 °C [38]. Rather than a random chain scission, thermal degradation occurs through a depolymerizing or “unzipping” mechanism that starts at the chain terminus, whereby the polymer chains undergo retro-polymerization to reform monomers (Scheme 6) [43,44,45,46,47].

Poly(CAs) are very susceptible to degradation following contact with water [41,44,47], but the rate of degradation is greatly accelerated under basic solutions [44,45]. Adventitious bases, present as impurities in common organic solvents, are sufficient to promote degradation [46]. Han and Kim stored a solution of poly(ethyl 2-cyanoacrylate) in acetone at room temperature and periodically tested the solution by gel permeation chromatography (GPC), demonstrating the polymer peak to gradually disappear with time [47]. Ryan and McCann showed the addition of tetrabutylammonium hydroxide (TBAOH) to poly(*n*-butyl cyanoacrylate) in THF at 21 °C deprotonated the chain end and led to a rapid depolymerization process [45]. The unzipped monomer is then instantly repolymerized in the presence of the base to form much lower molecular weight, so-called “daughter” polymers, in a depolymerization–repolymerization reaction. Similarly, solutions of *n*-butyl cyanoacrylate monomer in THF underwent instantaneous polymerization upon addition of millimolar quantities of TBAOH to give initially high molecular weight polymers, followed by rapid depolymerization and subsequent repolymerization to give lower molecular weight daughter polymers. This depolymerization–repolymerization reaction was confirmed by Robello et al. in their studies into the degradation of various poly(CAs) [46]. In this case the added base was not a necessity as degradation was observed in acetonitrile and acetone containing poly(CA) incubated at 50 °C. The degradation could be effectively inhibited by addition of acetic acid. As with the findings of the previous McCann and Ryan degradation study, the initial high molecular weight peak observed by GPC gradually disappeared over time and was accompanied by the simultaneous appearance of lower molecular weight peaks, indicating that the polymer chains are in dynamic equilibrium with their monomers.

## 3. Conventional (Non-Living) Radical Polymerizations

### 3.1. Inhibitors and Precautions 

Anionic polymerization is routinely inhibited by addition of parts per million of strong organic or mineral acids. These act by proton transfer to terminate anionic or zwitterionic species, before significant chain growth occurs. Anionic inhibitors have included Lewis acids, such as BF_3_ complexes [48], as well as, acetic acid [50,51,52,53], dichloroacetic acid [54], trifluoroacetic acid [55], methanesulfonic acid [47], and 1,3-propanesultone [51,56,57,58]. Excessive acidity should, however, be avoided, due to the potential for the hydrolysis of the monomer/polymer ester functionality leading to interference with the polymerization (see above).

Commercial CAs will often contain radical inhibitors, such as hydroquinone, catechol, *p*-methoxyphenol, butylated hydroxyanisole, and related phenolic compounds to suppress radical polymerization, which can be triggered with high temperatures or UV-light. In order to study the radical polymerization of CAs, inhibitors should be removed by distillation prior to commencing the reaction [58]. Any glassware that will come in contact with CAs should be acid washed, acetone rinsed, and oven dried before use. Distilled monomers should ideally be stored in tightly closed, high-density, polyethylene (HDPE) containers, at sub-zero temperatures. For solution polymerizations, attention should be paid to the inhibitor contained within commercial solvents which can often be nucleophilic in nature [46]. Similarly, any non-solvents used to isolate poly(CAs) by precipitation should be inhibited with strong acid (e.g., MeSO_3_H) to prevent anionic polymerization of residual monomers [58].

### 3.2. General Mechanism

**Scheme 7:**
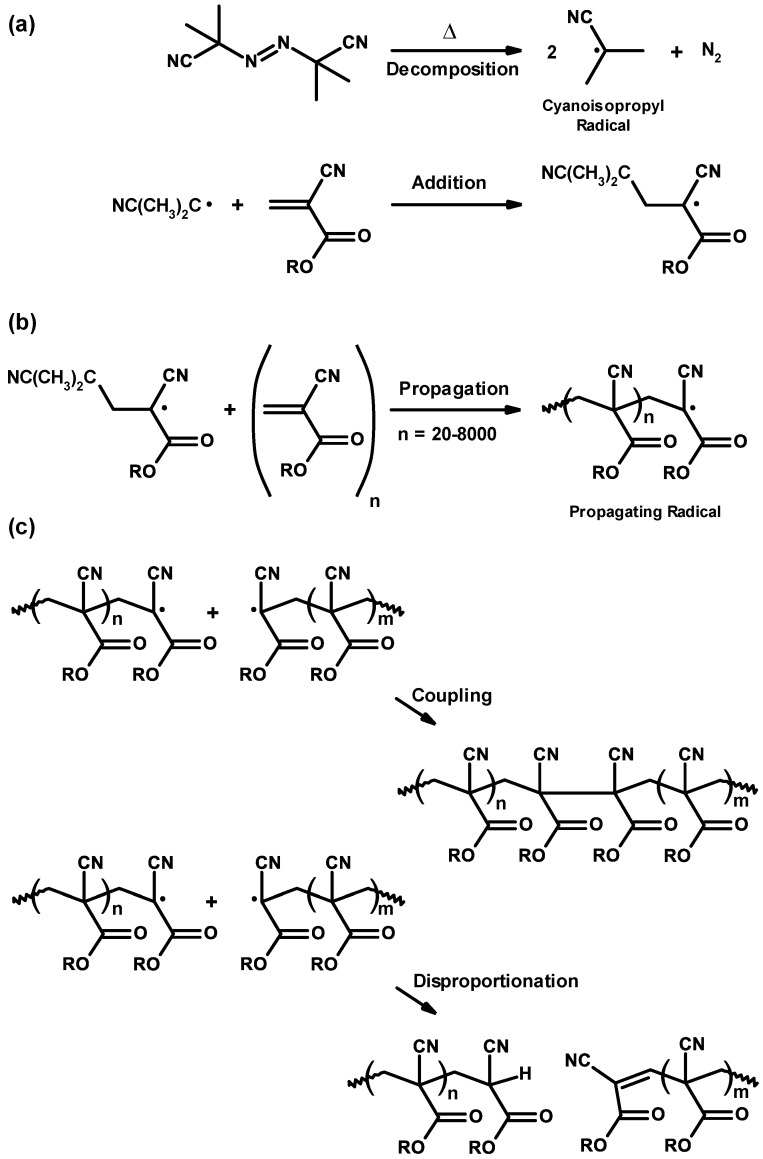
Conventional radical polymerization: (**a**); Initiation (**b**) Propagation; and (**c**) Termination.

Once the appropriate inhibitor for anionic polymerization is added, CAs can be polymerized radically (Scheme 7). Initiation takes place in two steps, as is normal in radical polymerization; the first involves the thermal homolysis of the initiator to give a pair of radicals. The initiator depicted is 2,2′-azobis(2-methylpropionitrile) (AIBN), an azo-initiator frequently used with CAs. After thermal decomposition into a pair of cyanoisopropyl radicals, the second step is the addition of the initiating radical onto the CA monomer to form a propagating radical. Rapid addition onto successive monomers occurs hundreds or even thousands of times, increasing the polymer chain length with each addition until terminating events take place. The process of initiation, propagation and termination take place for each propagating species, typically in the order of less than one second, or at most, a few seconds [59].

**Scheme 8:**
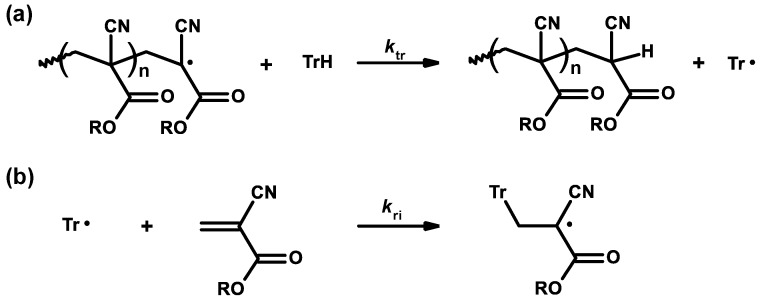
Chain transfer in radical polymerization: (**a**) Transfer and (**b**) Reinitiation.

There are two different means of termination: coupling and disproportionation. Termination by coupling occurs when two growing chains combine to give a single dead chain. Termination by disproportionation takes place when a β-hydrogen atom is abstracted from one propagating chain, resulting in a chain with a hydrogen terminus and the other with an unsaturated chain-end. An additional occurrence in radical polymerization is chain transfer reactions (*k*_tr_), whereby a propagating radical is terminated, usually by hydrogen atom abstraction, and a new radical species is created (Scheme 8). The newly created radical can then add to a monomer unit (*k*_ri_) and reinitiate polymerization. The chain transfer agent (TrH) can be the initiator, monomer, a solvent molecule or polymer.

### 3.3. Mechanism and Kinetics of Radical Homopolymerization

**Scheme 9:**
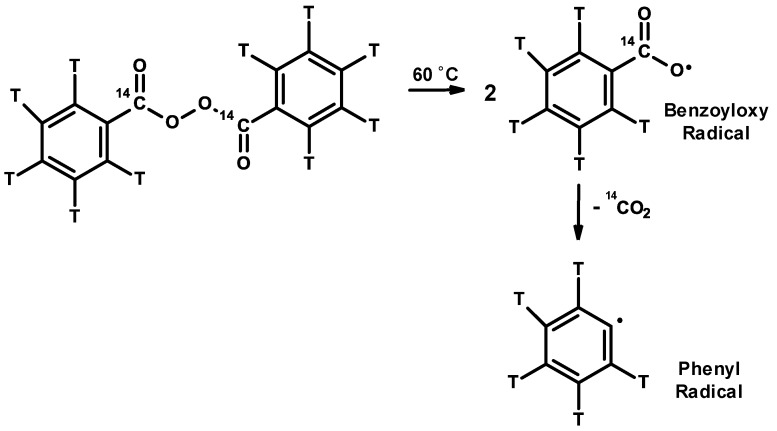
Initiation with doubly isotopically labelled benzoyl peroxide (BPO).

Various common azo- and peroxide radical initiators have been employed to initiate CA polymerization. AIBN and benzoyl peroxide (BPO) are the most frequently used, with the former being preferred. When BPO is used as a radical initiator, both benzoyloxy and phenyl radicals participate in initiation as the benzoyloxy radical decomposes to give a phenyl radical through loss of carbon dioxide (Scheme 9). Methyl 2-cyanoacrylate showed low reactivity towards the benzoyloxy radical, with initiation occurring largely through the addition of the more nucleophilic phenyl radical, as detected using doubly isotopically labelled BPO with carbon-14 and hydrogen-3 (tritium, T) [56]. For the polymerization at 60 °C, the number of phenyl α-end groups on the polymer was found to be approximately twice that of benzoyloxy end groups.

Chappelow et al investigated the use of tri-*n*-butyl borane oxide (TBBO) as the radical initiator for several highly reactive monomers: ethyl 2-isocyanatoacrylate, 2-isocyanatoethyl methacrylate, and ethyl 2-cyanoacrylate [60]. The initiating radical was generated from the auto-oxidation of the alkylboron compound with air to give the alkyl borane peroxide and butyl radicals [61]. Homopolymerizations were carried out using 1.8 wt% of TBBO in THF at room temperature. After 21 h, poly(ethyl 2-cyanoacrylate) was isolated by precipitation into hexane at greater than 70% conversion, however, polymer formation via anionic polymerization cannot be ruled out since no anionic inhibitor was employed.

The earliest report on the kinetics for the radical polymerization of CAs was by Canale et al. in 1960, who carried out polymerizations of methyl 2-cyanoacrylate using a boron trifluoride acetic acid complex as an anionic inhibitor and AIBN as the initiator [48]. The bulk parameter, *k*_p_/*k*_t_^0.5^, (where, *k*_p_ and *k*_t_ are the rate coefficients for propagation and termination, respectively) was determined to be 0.021 L·mol^−1^·s^−1^ at 60 °C. This was significantly greater than the *k*_p_/*k*_t_^0.5^ = 0.0144 L·mol^−1^·s^−1^ for MMA and *k*_p_/*k*_t_^0.5^ = 0.00166 L·mol^−1^·s^−1^ for styrene (St), measured in the same study, under the same conditions. Poly(methyl 2-cyanoacrylate) was found to be insoluble in common aromatic solvents, such as benzene and toluene. The homopolymer was also reported to be insoluble in alcohols, ketones (acetone, 2-butanone) and chlorinated solvents (chloroform, 1,2-dichloroethane).

Bevington et al. used 1,3-propanesultone (0.3 wt%) as an inhibitor in the radical polymerization of methyl 2-cyanoacrylate in bulk and in solutions of 1,4-dioxane at 60 °C using AIBN and BPO as initiators [57]. The inhibitor was reported to have negligible effects on the rate of polymerization at 60 °C, even at high concentrations, above 2.6 × 10^−2^ M. Although the polymer was found to be insoluble in dioxane, it was deemed an acceptable diluent for the polymerization at 60 °C. In a more recent study, looking at the preparation of highly branched CA containing polymers, the homopolymerization of ethyl 2-cyanoacrylate in two different solvents at 65 °C was carried out [55]. The polymerization was 50% faster in toluene than in acetonitrile due to an apparent poorer solubility of the polymer in the former, less polar solvent.

In 1983 Yamada et al. reported the first determination of absolute *k*_p_ and *k*_t_ for the radical polymerization of ethyl 2-cyanoacrylate using the rotating sector method [51]. The rotating sector method is so named as it refers to a rotating disc from which a sector-shaped portion is cut out; the disc is placed in between the reaction system and a non-laser light source to cause periodic interruption of light [62]. The cycling of light and dark periods allowed the parameter τ_s_, known as the average lifetime of a growing radical under steady-state conditions, to be calculated. Once τ_s_ is known, from equation 1, *k*_p_ and *k*_t_ values can be determined.

(1) $$\tau_{s}=\;\frac{k_{p}\left[M\right]}{2k_{t}\left(R_{p}\right)}$$

The values of *k*_p_ and *k*_t_ were determined for the bulk polymerization of ethyl 2-cyanoacrylate at 30 °C in the presence of two different anionic inhibitors. Using 7.0 wt% acetic acid, *k*_p_ = 1622 L·mol^−1^·s^−1^ and *k*_t_ = 4.11 × 10^8^ L·mol^−1^·s^−1^, and similar values of *k*_p_ = 1610 L·mol^−1^·s^−1^ and *k*_t_ = 4.04 × 10^8^ L·mol^−1^·s^−1^ were obtained when using 0.5 wt% 1,3-propanesultone. The close proximity of these values indicated that anionic polymerization was adequately suppressed by both inhibitors, and the rate coefficients were representative of the radical polymerization. The *k*_p_ values for ethyl 2-cyanoacrylate were close to those determined at 30 °C using the same method for ethyl 2-chloroacrylate and were attributed to the nitrile group attached to the α-carbon of the double bond having a similar stabilization effect on the propagating radical as the chlorine substituent at the same position [52]. Table 3 gives a comparison of experimentally measured *k*_p_ values using the rotating sector method. The *k*_p_ values for ethyl 2-cyanoacrylate are over five times higher than those measured for MMA and *n*-butyl methacrylate (*n*-BuMA) (Table 4). Yamada’s determination of *k*_p_ for the polymerization of ethyl 2-cyanoacrylate is in agreement with data derived from more recent density functional theory (DFT) computational modelling [63].

In 2015, the *k*_p_ for *n*-butyl 2-cyanoacrylate was estimated to be about seven times lower (*k*_p_ = 226 ± 32 L·mol^−1^·s^−1^) than that reported by Yamada et al for ethyl 2-cyanoacrylate [54]. The *k*_p_ was obtained by extrapolation of experimental data for the copolymerization of the CA with MMA using the more accurate pulsed-laser polymerization coupled with size exclusion chromatography (PLP-SEC) technique. PLP uses pulsed laser irradiation to oscillate between the light and dark periods; however, the pulse width is greatly shorter (nanoseconds) compared to the cycle time of the rotating sector method (seconds). The key advantage of PLP is that it allows for *k*_p_ to be calculated directly without the need to couple it to *k*_t_, and has led to greater reproducibility of kinetic results; compared to the discrepancies in experimental data observed for the rotating sector method. Consequently, PLP is now the International Union of Pure and Applied Chemistry (IUPAC) preferred method for *k*_p_ determination [64]. Table 4 gives a comparison of experimentally measured *k*_p_ for *n*-butyl 2-cyanoacrylate and other common monomers using PLP, and the reader is directed to benchmark Arrhenius parameters in authoritative reports [64,65]. It is difficult to draw direct comparisons between *k*_p_ values obtained from the rotating sector method and those obtained by PLP, since variations in experimentally reported *k*_p_ values from different studies can reflect differences in data interpretation and the dependence of kinetic parameters on polymerization conditions. The discrepancy in the *k*_p_ measured for the two different CA monomers by two different techniques is, however, significant, but, it is worth highlighting that the value obtained by Rooney et al. is an extrapolation rather than a direct PLP measurement [54].

As with radical polymerization of methacrylate monomers, termination in CA polymerizations is thought to predominantly occur by disproportionation. The hydrogen-atom terminated (non-functionalized) polymer chains are susceptible to the same degradation by base-catalyzed unzipping that occurs with anionically formed poly(CAs), where the chains with the unsaturated terminus do not unzip and are more stable [45,46]. Consequently, radically formed poly(CAs) exhibit greater stability than those formed by anionic polymerization. This improved stability was noted by Robello et al. during degradation studies of poly(CAs) [46]. Samples of poly(CA) derived from nucleophilic initiation completely degraded when incubated in acetonitrile at 50 °C for 24 h whereas only a small portion of samples derived from radical polymerization degraded under the same conditions. Manipulation of GPC data using a Gaussian numerical algorithm function for peak fitting enabled deconvolution of the GPC curves, which allowed an approximation that 80% of these polymer samples were terminated through disproportionation and 20% by combination.

### 3.4. Mechanism and Kinetics of Radical Copolymerization

**Scheme 10:**
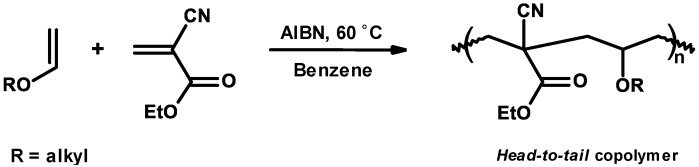
Radical copolymerization of ethyl 2-cyanoacrylate and vinyl ether.

One key benefit of radical polymerization, which is difficult to achieve through anionic means, is the ease of copolymerization with other monomers, generating polymers with unique and varying properties. CAs form alternating head-to-tail copolymers when radically copolymerized with electron-rich monomers, such as vinyl ethers (Scheme 10) [69,70].

For a vinyl monomer, M_1_, being copolymerized with monomer M_2_, the reactivity ratios, r_1_ and r_2_, represent the tendency of the propagating chain-end species to self-propagate with its own monomer over that of the other monomer. Although it has been met with criticism, historically, the *Q* and *e* scheme has been used as a measure of monomer and radical reactivity on a quantitative basis, in terms of correlating structure with reactivity, and can be used to predict reactivity ratios [71,72]. The *Q* and *e* values are related to the extent of resonance stabilization and polarity of the monomer and are approximated from its copolymerization kinetic data, whereby the parameter *Q* describes the resonance factor (and to a certain degree the steric factor) of the monomer and the parameter *e* describes the polar factor. *Q* and *e* values assigned to monomers are correlated against the arbitrarily selected reference values of *Q* = 1 and *e* = −0.80 for St. The values of *Q* and *e* increase with increasing monomer reactivity and increasing electron deficiency of the carbon–carbon double bond. Negative values of *e* indicate an electron-rich double bond. The values for methyl 2-cyanoacrylate were reported by Otsu and Yamada as *Q* = 17 and *e* = 2.48 from the bulk copolymerization with MMA in the presence of acetic acid in a sealed tube at 60 °C using AIBN as the initiator [50]. The authors noted that the monomers showed a high tendency for alternation, with rates of copolymerization decreasing as the CA monomer concentration increased. The modes of derivation for *Q* and *e* values without the necessity for an arbitrary assignment or without equating the polarities of conjugate monomers and radicals have led to improved methods of assessment for reactivity ratios [73,74]. Recent values of *Q* and *e* for methyl 2-cyanoacrylate have been reported as 4.91 and 0.91, respectively [75]. The values of *Q* and *e* for some common monomers are shown in Table 5.

Kinsinger et al. examined the radical copolymerization of methyl 2-cyanoacrylate with a wide variety of monomers [76]. The reactivity ratios for methyl 2-cyanoacrylate (M_1_) with a number of reference monomers in benzene at 60 °C using AIBN as the initiator were determined, the most important of which was that of MMA (M_2_), reported as r_1_ = 0.25 and r_2_ = 0.04, given that MMA has been one of the more frequently studied co-monomers with CAs. In 2016, the reactivity ratios of ethyl 2-cyanoacrylate and MMA were simulated on PROCOP software by Tang and Tsarevsky, based on their experimental copolymerization data in toluene at 65 °C [55]. The terminal kinetic model was used, and the theoretical kinetic curves were fitted to the experimental data. The values reported were r_1_ = 0.15 and r_2_ = 0.02, which are not too dissimilar to those reported by Kinsinger. Over the years, a series of reactivity ratios for CAs with a variety of monomers have been reported, which are summarized in Table 6.

Han and Kim carried out extensive studies on the stability and degradation of copolymers from the radical copolymerization of ethyl 2-cyanoacrylate and MMA using AIBN at 60 °C with MeSO_3_H as an anionic inhibitor [47]. These copolymers were found to be random in nature with a strong alternating tendency. Due to this alternation in the copolymer system, the inclusion of MMA units in the polymer backbone close to the chain terminus was believed to be the reason for suppressing the unzipping degradation of the polymer. The copolymers were found to have increasing thermal stability with increasing MMA content.

**Scheme 11:**
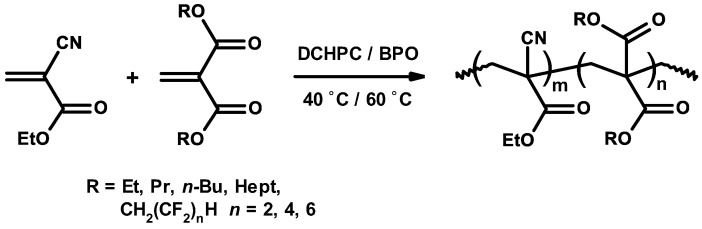
Copolymerization of CA with alkyl/fluoroalkyl methylene malonates.

Like CAs, methylene esters of malonic acid are a group of electron deficient monomers which can be polymerized both anionically and radically. Polyakova et al. studied the anionic and radical copolymerization of a series of alkyl and fluoroalkyl methylene malonates with ethyl 2-cyanoacrylate using a molar feed of 5–50% [77]. Bulk anionic copolymerizations were carried out using a *tert*-BuLi as the initiator at 20 °C, whereas bulk radical copolymerizations were carried out using dicyclohexyl peroxydicarbonate (DCHPC) and BPO at 40 °C and 60 °C, respectively, to generate random copolymers (Scheme 11). The fluoroalkyl methylene malonates were observed to have the highest reactivity toward ethyl 2-cyanoacrylate, as determined by elemental analysis. The mechanical and chemical properties of cured poly(CA) based adhesive formulations containing 10% of alkyl and fluoroalkyl methylene malonates were tested. Formulations containing fluoroalkyl methylene malonate were found to have improved thermal and hydrolytic stability, compared to the control formulation based on ethyl 2-cyanoacrylate alone.

Kinsinger et al. detailed a lack of success in attempting to copolymerize 1-octene with methyl 2-cyanoacrylate, which largely resulted in CA homopolymer being formed [76]. Sperlich and Eisenbach overcame this issue by employing CF_3_CO_2_H or ZnCl_2_.OEt_2_ as complexing agents in the copolymerization of ethylene with ethyl 2-cyanoacrylate to form alternating copolymers [78]. Copolymerizations were carried out in a pressure reactor using DCHPC as initiator, with reaction components added together at −80 °C before being heated to 40 °C and maintained at that temperature for 15 h. The concentration of complexing agent was found to have a significant influence on the degree of alternation of the copolymer.

Dikov et al. reported on the radical graft copolymerization of ethyl 2-cyanoacrylate onto poly(butadiene-*co*-AN) using bulk polymerizations at 60–80 °C in the presence of up to 2 wt% of the dissolved polymer with BPO as the initiator and TsOH (1 wt%) as an anionic inhibitor [79]. Graft polymerizations are useful as they allow combination of two polymers which would otherwise be incompatible. Solution copolymerizations were also carried out in toluene at 80–90 °C with up to 75 wt% poly(butadiene-*co*-AN) present. IR spectra of the resulting polymers gave superposition of the individual polymer spectra and were considered a good indication of a successful graft copolymerization.

Polymers can act as piezoelectric materials whereby polymers’ functionality can repel or attract each other when an electrical field is applied. Hall Jr et al. synthesized a large number of nitrile containing copolymers and studied their piezoelectric properties in terms of their pyroelectric coefficients [80]. Copolymers of CA with vinyl acetate (VAc) and isopropenyl acetate were prepared using AIBN in benzene at 60–65 °C with 7 wt% of acetic acid. A copolymer of ethyl 2-cyanoacrylate and VAc gave a value of *p* = 6.7 μC·m^−^2·K^−1^, one of the highest values recorded in the study, in contrast to the value of *p* = 1.94 μC·m^−2^·K^−1^ measured for a copolymer of acrylonitrile (AN) and VAc.

**Scheme 12:**
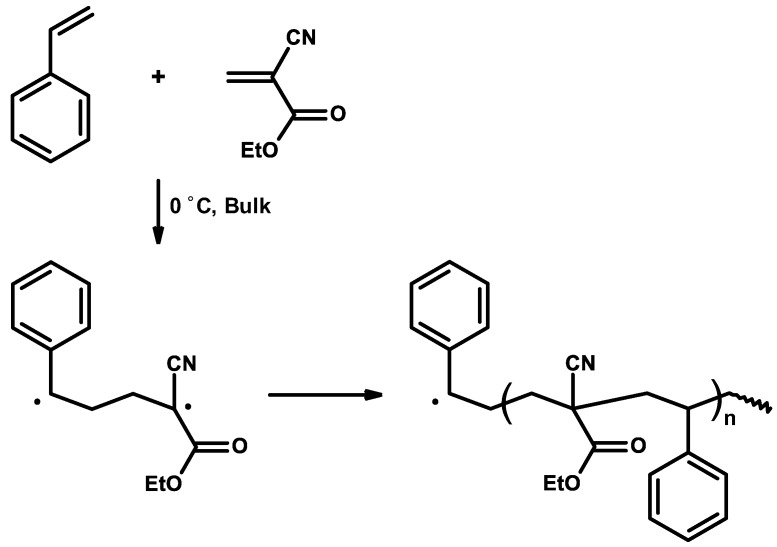
Spontaneous copolymerization of St and ethyl 2-cyanoacrylate.

When CAs are added directly to certain electron-rich vinyl monomers, spontaneous copolymerization can occur without the requirement of an external initiator, even at room temperature—this is believed to occur through the formation of diradical intermediates (Scheme 12) [81,82]. After the formation of the intermediate diradical species, the electron-deficient CA radical rapidly adds onto another St monomer to form a new benzylic radical, which, in turn, further reacts with an CA monomer, and an alternating copolymer is generated.

### 3.5. Synthetic Copolymerization Studies

**Scheme 13:**
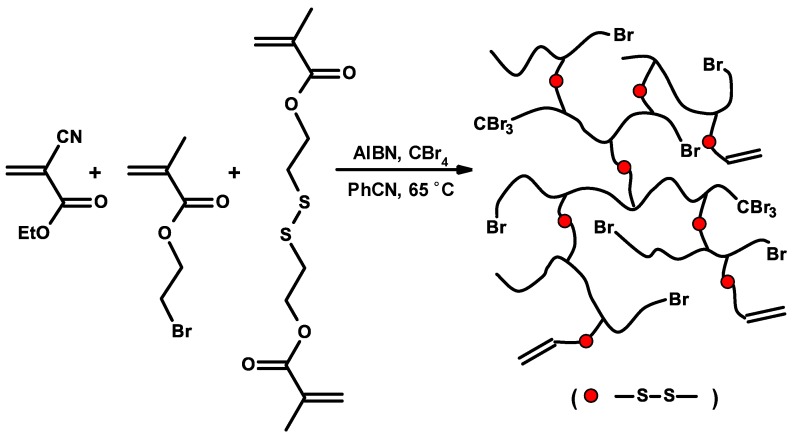
Preparation of disulfide-containing branched CA-based copolymers.

Branched ethyl 2-cyanoacrylate copolymers were recently reported using small amounts of haloethyl methacrylates in the presence of CBr_4_ and *bis*(2-methacryloyloxyethyl) disulfide ((MAOE)_2_S_2_), a disulfide-based dimethacrylate crosslinker (Scheme 13) [55]. Highly branched poly(CAs) are of special interest due to unique characteristics, like enhanced solubility and reduced viscosity, in comparison to their linear analogues with the same molecular weight. AIBN was used as the initiator at 65 °C, CF₃CO₂H as an anionic inhibitor and CBr_4_ was employed as a chain transfer agent to limit the polymer chain growth, inhibit gelation, and provide bromide functionality to the chain ends. Typical relative ratios were [AIBN]_0_:[CBr_4_]_0_:[ECA]_0_:[HaloEMA]_0_:[(MAOE)_2_S_2_]_0_ = 1:200:500:300:100, and by varying the amount of chain-transfer agent, crosslinker and comonomer, the point of gelation was modified for different conversions and reaction times. The resulting highly branched copolymers were selectively degraded by treatment with Bu_3_P to reductively cleave the disulfide bridges.

### 3.6. Nanoparticles

**Figure 2:**
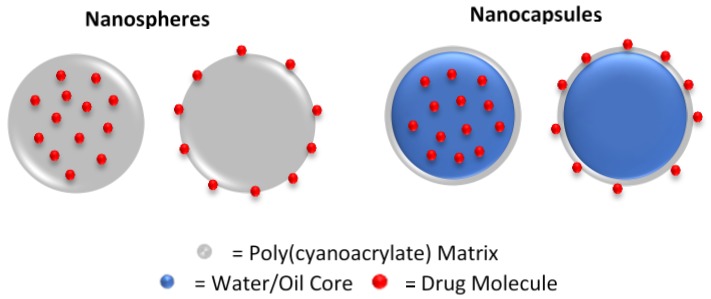
Types of nanoparticles.

Colloidal nanosystems for drug delivery alter the pharmacokinetics of numerous drugs, which can often improve their therapeutic indexes [83]. These colloids are usually spherical, submicron in size, stable in bodily fluids, biodegradable, and have no systematic toxicity or immune response. Ideally, these nanoparticles can be loaded with various drugs and targeted to a specific location in the body, allowing drugs to be delivered to certain organs or cells but not to others. Site-specific targeting gives increased drug concentration to infected or abnormal cells and low concentration to normal cells, thus decreasing drug toxicity and undesirable side effects. Many synthetic polymers, such as poly(lactic acid) and poly(caprolactone), have found utility in the preparation of various drug nanocarriers, with poly(CA) becoming an established colloidal delivery system, since its introduction almost 40 years ago [84]. Poly(CA) is a favoured nanocarrier due to its ease of polymerization, biodegradability, and relatively low toxicity. Broadly speaking, polymeric nanoparticles used for drug delivery are either solid particles or hollow particles, and can be prepared by a wide variety of methods. In the case of solid particles, the drug would typically be distributed throughout the particle or located at/near the interface. In the case of hollow particles (capsules), the drug is normally solubilized in a liquid core of either water or oil, surrounded by a polymer shell (Figure 2).

Poly(CA) nanospheres are classically prepared by anionic emulsion or mini-emulsion polymerizations of the monomer in an acidic aqueous medium, typically of pH ≈ 3, containing a surfactant as a colloidal stabilizing agent. This relatively simple process was first reported by Couvreur et al. in 1979 [84], and numerous studies have investigated the polymerization parameters and kinetics [85,86,87]. Alternatively, poly(CA) nanospheres can be produced by radical polymerization using traditional radical initiators, such as AIBN, though at a very low pH of close to 1 [88]. These radical mini-emulsion polymerizations have the advantage of achieving high molecular weight poly(CAs), whereas, in the case of anionic polymerization, the molecular weight is strongly dependent on the pH of the medium and typically results in polymers below 8000 g·mol^−1^. Typically, nanoparticles have a hydrophilic shell based on poly(ethylene oxide, PEO) and hydrophobic core, based on poly(*n*-butyl 2-cyanoacrylate). [88,89] PEO can not only impart hydrophilicity on polymers, but modify toxicity (see below).

**Scheme 14:**
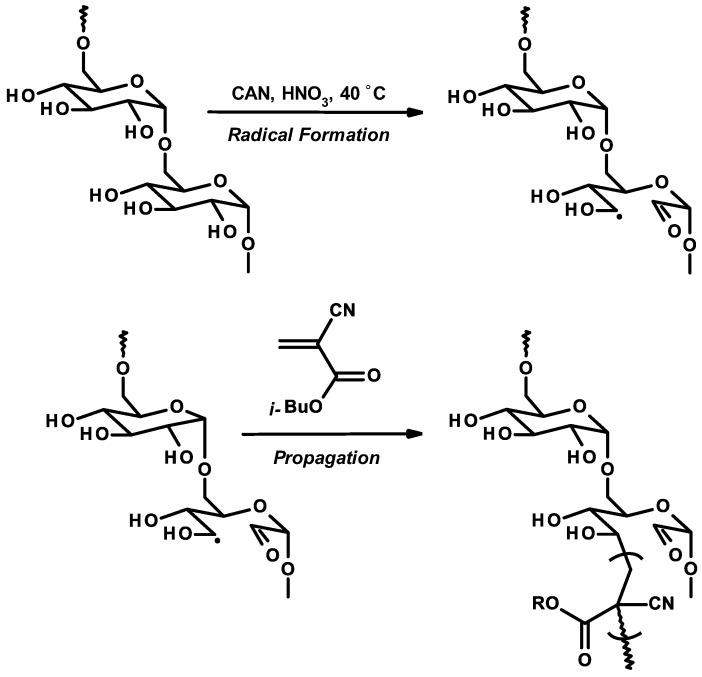
CAN-initiated radical polymerization of CA.

Chauvierre et al. employed redox radical polymerization using cerium ammonium nitrate, (NH₄)₂Ce(NO₃)₆, (CAN) in HNO_3_ to initiate polymerization in the presence of various polysaccharides, such as dextran, heparin or chitosan [90]. Initiation and propagation of isobutylcyanoacrylate (*i*-BuCA) occurred as the monomer was added to the polysaccharide and CAN mixture to form the polysaccharide-poly(*i*-BuCA) copolymer (Scheme 14). The rate of polymerization was found to be high due to the fast initiation step. The high radical initiation rate at a low pH renders any anionic polymerization negligible within the timescale of the reaction and allows radical propagation to predominate. This initiation process has since been applied to emulsion polymerizations in the preparation of poly(CA)-based nanospheres with various polysaccharides on the surface [91,92].

**Scheme 15:**
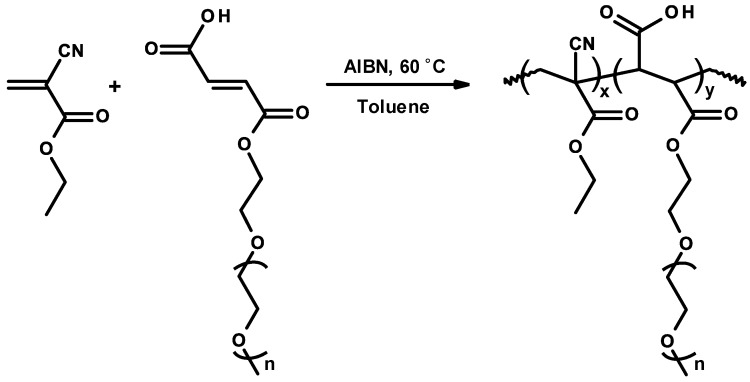
Polymerization of a PEG macromonomer with ethyl 2-cyanoacrylate.

**Figure 3:**
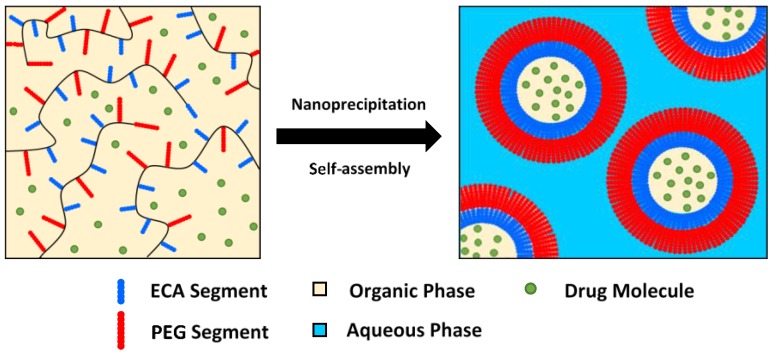
Formation of nanocapsules by polymer self-assembly.

Poly(CA) nanocapsules are typically prepared by anionic interfacial polymerization techniques in water-in-oil or oil-in-water emulsion systems [93,94]; however, nanocapsules can also be formed by nanoprecipitation. Based on the nanoprecipitation method, preformed polymers (such as those made by radical polymerization) are dissolved in an organic solvent, typically acetone, and then added dropwise to an aqueous solution of surfactant where self-assembly gives nanocapsules [95]. Using this approach, poly[α-maleic anhydride-ω-methoxypoly(ethylene glycol, PEG)-*co*-EtCA] copolymers were prepared by radical solution copolymerization of a PEG macromonomer with ethyl 2-cyanoacrylate at 60 °C using AIBN (Scheme 15) [96]. The polymer formed by precipitation was added to acetone containing the drug (ibuprofen), which successfully self-assembled upon addition to water to encapsulate the drug (Figure 3) [97]. PEGylated particles (also termed “stealth” nanoparticles) are of great significance as they can escape immuno-recognition to give long-circulating drug delivery vehicles [98,99].

Nanoparticles based on CAs are considered promising polymer colloidal drug delivery systems, and have been significantly studied for cancer therapy with certain formulations reaching Phases III in clinical trials [100].

### 3.7. Controlled/Living Radical Polymerizations

**Scheme 16:**
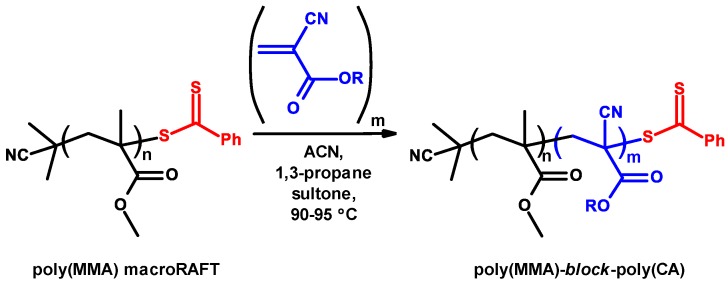
RAFT polymerization of CAs using a macroRAFT (reversible addition-fragmentation chain transfer) agent.

There has been only one report on controlled/living radical polymerization of CAs, which used reversible addition-fragmentation chain transfer (RAFT) [58]. RAFT is perhaps the most versatile reversible deactivation (controlled/living) radical polymerization technique for making precision polymers [101]. Given that CA propagates via a tertiary radical, poly(MMA), a reasonably bulky macroRAFT agent was used to make the first block copolymers containing poly(CAs) (Scheme 16). ACN (1,1’-azobis(cyclohexanenitrile)) and 1,3-propanesultone were used as the respective radical initiator and anionic stabilizer at 90–95 °C.

**Scheme 17:**
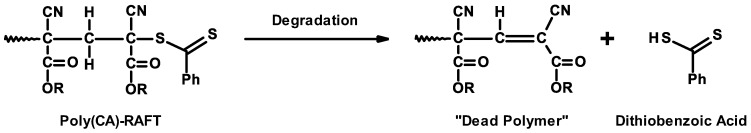
Elimination of the RAFT end group.

Controlled/living character in CA polymerizations were indicated by a shift in the molecular weight distributions (MWDs) to higher MW with increasing conversion, with the MWD remaining mono-modal and relatively narrow throughout. However, the refractive index (RI) GPC MWDs disguised an underlying problem, which was the inherent instability of the CA block copolymers through self-elimination of the RAFT end group to generate dithiobenzoic acid (Scheme 17). The degradation of the RAFT end group, which was not apparent by RI detection of GPC, could be visualized by UV analysis of polymer samples. UV showed several low MW peaks that were absent in the RI data, which increased in intensity with increasing conversion.

It seems that the acidity of the methylene at the chain terminus is enhanced by the inductively electron-withdrawing nitrile and ester functionalities, leading to the formation of an unsaturated end group. The polymerization is then complicated by the many possible combinations and disproportionation reactions between propagating and/or dithiobenzoic acid-derived radicals, which account for the observed increase in the number of polymer chains obtained through analysis of GPC RI data. For all three CAs investigated (ethyl 2-cyanoacrylate, *n*-butyl 2-cyanoacrylate and phenylethyl 2-cyanoacrylate), loss of livingness was about 70% by the end of the polymerizations.

## 4. Conclusions

Over the years researchers have found the instant adhesive action of CAs to be a significant impediment when it comes to realizing their synthetic potential. Clearly, this is a challenging monomer class for radical homopolymerization, with disputed polymerization rate coefficients. Copolymerization via radical means is facile, in contrast to anionic copolymerization, and the derived copolymers are more stable due to suppression of the unzipping process. The greater stability over polymers made by the anionic pathway is due to the greater preponderance of unsaturated end groups, and the incorporation of alternative monomer repeat unit using radical polymerization. Alternating copolymers are formed by CA radical copolymerizations with electron-rich and non-activated monomers, with initiator-free spontaneous copolymerizations also possible. Copolymers of CAs with electron-rich vinyl monomers offer superior piezoelectric properties, compared to analogous copolymers with AN. RAFT enabled the preparation of the first block copolymers containing poly(CA), although control/living character was limited by the inherent instability of the resultant polymer chains that eliminate the RAFT end group. Nevertheless, the low toxicity and biocompatibility of poly(CA) materials provides stimulus for continued research, especially in the area of controlled drug release. Particularly notable are nanoparticle formations via mini-emulsion radical copolymerizations with PEG.

## Figures and Tables

**Table 1:** Structure and commercial uses of common cyanoacrylates (CAs).

Structure	R	Uses
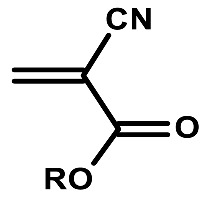	Me	Loctite 496 super bonder
	Et	Loctite 401 instant adhesive
	*i*-Propyl	Root canal sealant
	*n*-Butyl	Skin/surgical adhesive
	2-Octyl	Skin surgical adhesive
	Allyl	High temperature adhesive
	β-Methoxyethyl	Low odor adhesive
	β-Ethoxyethyl	Low odor adhesive
	Phenylethyl	Adhesive tapes

**Table 2:** Glass transition temperatures (*T*_g_) of poly(CAs).

Poly(CA)	*T*_g_, °C	Ref.
MeCA	160	[36]
EtCA	150	[38]
*n*-BuCA (isobutylcyanoacrylate)	130	[37]
2-OctylCA	10	[1]
AllylCA	90	[37]
β-MethoxyethylCA	85	[1]

**Table 3:** Propagation rate coefficient (*k*_p_) values at 30 °C using the rotating sector method.

Monomer	*k*_p_ (L·mol^−1^·s^−1^)	Ref.
EtCA	1610–1622	[51]
Ethyl chloroacrylate	1660	[52]
Ethyl fluoroacrylate	1120	[53]

**Table 4:** *k*_p_ values using the pulsed-laser polymerization coupled with size exclusion chromatography (PLP-SEC) method of *n*-butyl cyanoacrylate at 30 °C and critically evaluated *k*_p_ values of common vinyl monomers at 20 °C.

Monomer	*k*_p_ (L·mol^−1^·s^−1^)	Ref.
*n*-BuCA ^a^	226 ± 32	[54]
methyl methacrylate	270	[66]
*n*-BuMA	310	[67]
MA	15,851	[65]
St ^a^	107	[68]

^a^ Measured at 30 °C.

**Table 5:** *Q* and *e* values for methyl 2-cyanoacrylate (MeCN) and common monomers.

Monomer	*Q*	*e*
MeCA	4.91	0.91
Vinyl Acetate (VAc)	0.026	−0.88
St	1.00	−0.80
Isoprene	1.99	−0.55
1,3-Butadiene	1.70	−0.50
MMA	0.78	0.40
Acrylamide	0.23	0.54
MA	0.45	0.64
Ethyl vinyl ether	0.018	−1.80
Acrylonitrile (AN)	0.48	1.23

**Table 6:** Reactivity ratios of CAs with vinyl monomers at 60 °C.

CA (M_1_)	Monomer (M_2_)	r _1_	r _2_	Medium	Ref.
MeCA	MA	1.2	0.1	Benzene	[76]
MeCA	MMA	0.25	0.04	Benzene	[76]
MeCA	St	0.03	0.01	Benzene	[76]
MeCA	α-Methyl St	0.001	0.05	Benzene	[76]
MeCA	VAc ^c^	0.5	0.005	Benzene	[76]
MeCA	MMA	0.13	0.10	Bulk	[50]
EtCA	MMA	0.85	0.41	Bulk	[47]
EtCA	MMA	0.16	0.08 ^a^	Bulk	[51]
*n*-BuCA	MMA	0.236	0.057 ^b^	Bulk	[54]

^a^ Measured at 30 °C, ^b^ Measured at 50 °C, ^c^ VAc is vinyl acetate.
